# Assembly and comparative analysis of the first complete mitochondrial genome of *Salix psammophila*, a good windbreak and sand fixation shrub

**DOI:** 10.3389/fpls.2024.1411289

**Published:** 2024-10-02

**Authors:** Hongxia Qiao, Yajuan Chen, Ruiping Wang, Wei Zhang, Zhang Zhang, Fengqiang Yu, Haifeng Yang, Guiming Liu, Jiewei Zhang

**Affiliations:** ^1^ Beijing Academy of Agriculture and Forestry Sciences, Beijing, China; ^2^ Beijing Key Laboratory of Agricultural Genetic Resources and Biotechnology, Institute of Biotechnology, Beijing, China; ^3^ College of Forestry, Inner Mongolia Agricultural University, Hohhot, China; ^4^ Ordos Forestry and Grassland Development Center, Ordos, China

**Keywords:** *Salix psammophila*, Salicaceae, mitochondrial genome, Pacbio HiFi, comparative analysis

## Abstract

*Salix psammophila*, commonly known as the sandlive willow, is a vital shrub species within the Salicaceae family, particularly significant for its ecological role in regions susceptible to desertification and sandy soils. In this study, we assembled the complete *S. psammophila* mitochondrial genome using Pacbio HiFi third-generation sequencing data. The genome was found to be a typical single circular structure, with a total length of 715,555 bp and a GC content of 44.89%. We annotated 33 unique protein-coding genes (PCGs), which included 24 core mitochondrial genes and 9 variable genes, as well as 18 tRNA genes (5 of which were multicopy genes) and 3 rRNA genes. Comparative analysis of the PCGs from the mitochondrial genomes of *S. psammophila*, *Populus deltoides*, *Populus simonii*, *Salix wilsonii*, and *Salix suchowensis* revealed that these genes are relatively conserved within the Salicaceae family, with variability primarily occurring in the ribosomal protein genes. The absence of the *rps14*, which encodes a ribosomal protein, may have played a role in the evolution of stress tolerance in Salicaceae plants. Additionally, we identified 232 SSRs, 19 tandem repeat sequences, and 236 dispersed repeat sequences in the *S. psammophila* mitochondrial genome, with palindromic and forward repeats being the most abundant. The longest palindromic repeat measured 260 bp, while the longest forward repeat was 86,068 bp. Furthermore, 324 potential RNA editing sites were discovered, all involving C-to-U edits, with the *nad4* having the highest number of edits. These findings provide valuable insights into the phylogenetic and genetic research of Salicaceae plants.

## Introduction

*Salix psammophila* is a perennial fast-growing and multi-resistance shrub in the genus Salix of the Salicaceae family, naturally distributed in arid and semi-arid desert areas, produced in the Maowusu Sandland and the Kubuqi Desert, and is the first choice of species for the prevention and control of desertification areas (Bao and Zhang, 2012; Jia et al., 2019; Lu et al., 2019; Yang et al., 2022). In the *S. psammophila* asexual line, the 17–38 asexual line is an excellent asexual variety, and in the morphology of the performance of the upright, short branchlets, the landscape effect is good. Currently, research on *S. psammophila* mainly focuses on strain type and sand barriers, while the genetic resources and phylogenetic status of *S. psammophila* mitochondria are still unclear. In order to further develop and utilize *S. psammophila*, the 17–38 asexual line of *S. psammophila* was used as a germplasm resource to assemble and annotate the *S. psammophila* mitochondrial genome and to analyze the structural characteristics and phylogenetic status of the *S. psammophila* mitochondrial genome.

The mitochondria are organelles with semi-autonomous genetic characteristics, relatively independent of the nucleus, with a relatively independent genetic system (Gray, 2012; Chen et al., 2017). It is an important organelle in eukaryotic cells that produce ATP by oxidative phosphorylation and is a source of energy for a variety of biochemical processes, playing a crucial role in plant development and reproduction (Wu et al., 2019). Plant mitochondrial genomes have a variety of structures due to the possession of a large number of repetitive sequences, and assembled mitochondrial genomes may be unicyclic, polycyclic, linear, or multibranching in structure (Ye et al., 2017; Cheng et al., 2021). For example, the mitochondrial genome of *Populus deltoides* and *P. simonii* has been assembled into three circular chromosomes. In contrast, the mitochondrial genome of *P. tremula* and *P. tremula* × *P. alba* is each organized into a single circular chromosome (Kersten et al., 2016; Bi et al., 2022; Qu et al., 2022). In addition, the size of plant mitochondrial genomes varies greatly, for instance, the small 66-kb *Viscum scurruloideum* (mistletoe) and the large 11.3-Mb *Silene conica* (Sloan et al., 2012; Skippington et al., 2015). It has been shown that the main cause of mitochondrial genome amplification in plants is the insertion of foreign sequences and the presence of a large number of repetitive sequences (Richardson and Palmer, 2006; Li, 2009; Smith, 2011). The number of genes in plant mitogenomes also varied, from 19 in the *Viscum album* to 221 in the pepper (*Capsicum annuum*) (Jo et al., 2011; Petersen et al., 2015).

Although the size, structure, and number of plant mitogenomes vary greatly, the encoded genes are relatively conserved, such as ATP synthase genes, NADH dehydrogenase genes, cytochrome C reductase genes, and cytochrome C oxidase genes (Bi et al., 2020). In addition, there are protein-coding genes (PCGs) that are lost during the evolution of the plant mitochondrial genome, such as the succinate dehydrogenase genes and the ribosomal protein (RP) genes (Bi et al., 2016). The genes *rps12*, *sdh3*, and *sdh4* appear to be absent in the monocotyledonous plants, whereas the *rps2* gene is notably missing in dicotyledonous plants (Zhang et al., 2012). RPs have important roles in metabolism, cell division, and growth processes (Wang et al., 2013a). RP genes are among the most highly expressed genes in most cells, and their expressions are differentially regulated by environmental and signaling molecules (Vemanna et al., 2019; Petibon et al., 2020; Fakih et al., 2023). Wang et al. explored the changes of ribosomes in phosphate- and iron-deficient *Arabidopsis* roots and found that most RP genes were related to translation and ribosome assembly, and some were related to low temperature, UV-B, and salt stress (Wang et al., 2013a).

It is well known that plant mitochondrial genomes vary greatly in size, structure, incorporation of exogenous DNA, and mutation rates, and there are a large number of repetitive sequences in the genomes, making it difficult to sequence plant mitochondrial genomes (Petersen et al., 2020). However, with the rapid development of third-generation sequencing technology, especially the emergence of PacBio and Oxford Nanopore, more and more plant mitochondrial genomes have been assembled and submitted to the NCBI database. In this study, we obtained, assembled, and annotated the complete *S. psammophila* mitochondrial genome for the first time based on Pacbio HiFi third-generation sequencing data. Characteristics of the *S. psammophila* mitochondrial genome were analyzed, including codon preference, RNA editing events, repetitive sequence analysis, phylogenetic analysis, and sequence migration analysis. These results will further lay the foundation for understanding and studying the evolution and inheritance of mitochondria in Salicaceae plants.

## Materials and methods

### Plant materials, DNA extraction, and sequencing

The experimental material used in this study was mature leaves of the 3-year-old 17–38 asexual clone of *S. psammophila* from the Germplasm Resources gene bank of *S. psammophila* in Ordos Dalad, the Inner Mongolia Autonomous Region of China (E 110°38′59.1″, N 40°14′15.5″). The leaves were collected, snap frozen in liquid nitrogen, and stored in a −80°C refrigerator. The total genomic DNA was extracted using a plant DNA extraction kit and sequenced with Pacbio HiFi sequencing technology following the manufacturer’s kits and protocols (Zhang et al., 2012, 2024).

### Assembly and annotation of the mitochondrial genome

We used Pacbio HiFi third-generation sequencing data to assemble the *S. psammophila* mitochondrial genome. The long-reads data were assembled using the default parameters of the Flye software (v2.9.4) (Kolmogorov et al., 2019) to obtain a graphical mitochondrial genome in GFA format. Use makeblastdb to build a library of all fasta-formatted contigs assembled. Then, the contig fragments containing the mitochondrial genome were identified using the BLASTn program (Chen et al., 2015) and using the plant mitochondrial genome from *Arabidopsis thaliana* as a reference genome with the parameters “-evalue 1e-5 -outfmt 6 -max_hsps 10 -word_size 7 -task blastn-short”. In addition, we also included two closely related species, *S. wilsonii* and *S. suchowensis*, as references for re-assembly. This mitochondrial genome was visualized by Bandage software (v0.8.1) (Wick et al., 2015) and screened for mitochondrial contigs based on BLASTn results to obtain a sketch of the mitochondrial genome of *S. psammophila*.

*A. thaliana* (NC_037304), *Liriodendron tulipifera* (NC_021152.1), *P. simonii* (MZ905370), and *S. wilsonii* (NC_064688.1) were used as reference genomes, and the mitochondrial genome of *S. psammophila* was annotated using Geseq software (v2.03) (Tillich et al., 2017). The tRNA and rRNA of the genome were annotated using Tr4Nascan-SE software (v.2.0.11) (Lowe and Eddy, 1997) and BLASTn software (v2.13.0) (Chen et al., 2015). Finally, each mitochondrial genome annotation was manually corrected using Apollo software (v1.11.8) (Lewis et al., 2002).

### Structural analysis and codon preference analysis of the genome

To resolve the obtained repetitive regions in the graphical mitochondrial genome, the long reads were compared to the repetitive sequences to determine whether any long reads crossed the repetitive region, especially the *ctg3* repetitive region. Selection of suitable pathways is based on long reads to hypothesize the most likely mitochondrial genome structure formed in *S. psammophila*.

Genomic PCGs were extracted by the Phylosuite software (v1.1.16) (Zhang et al., 2020) and then analyzed for codon preference by the Mega software (v7.0) (Kumar et al., 2016), and RSCU values were calculated.

### Sequence repeat analysis

Microsatellite repeats, tandem repeats, and dispersed repeats were identified using MISA (v2.1) (https://webblast.ipk-gatersleben.de/misa/) (Beier et al., 2017), TRF (v4.09) (https://tandem.bu.edu/trf/trf.unix.help.html) (Benson, 1999), and the REPuter web server (https://bibiserv.cebitec.uni-bielefeld.de/reputer/) (Kurtz, 2001), and the results were analyzed using Excel software and the Circos package (v0.69.9) (Zhang et al., 2013) for visualization.

### Sequence transfer analysis and synteny analysis

The chloroplast genome was assembled and annotated using the GetOrganelle software (Jin et al., 2020) and CPGAVAS2 software (Shi et al., 2019). The annotation results were subsequently corrected using the CPGView software (Liu et al., 2023). Homologous fragments of the mitochondrial and chloroplast genomes were analyzed using the BLASTn software (v2.13.0) (Chen et al., 2015), and the results were visualized using the Circos package (v0.69.9) (Zhang et al., 2013).

Six tree species, including *S. psammophila*, were selected for analysis, and based on the BLASTn results of two–two comparisons between individual mitochondrial genomes, homologous sequences of more than 500 bp were retained as conserved covariate blocks, and Multiple Synteny Plot was plotted.

### Prediction of RNA editing sites

We used the sequences of all PCGs encoded by the *S. psammophila* mitochondrial genome as the input file and used Deepred-mt (Edera et al., 2021) to predict RNA editing sites from C to U of the mitochondrial PCGs, and retained all results with probability values greater than 0.9.

### Phylogenetic analysis

Thirty-four mitochondrial genomes from three families, namely, Salicaceae, Euphorbiaceae, and Rhizophoraceae, were selected for phylogenetic analysis based on their affinities, with two Rhizophoraceae mitochondrial genomes set as the outgroup. Common genes were extracted using the PhyloSuite software (v1.1.16) (Zhang et al., 2020). Multiple sequence alignment was performed using MAFFT software (v7.505) (Katoh and Standley, 2013). Then, phylogenetic trees were constructed using the IQ-TREE software (v1.6.12) (Nguyen et al., 2015) based on the maximum likelihood (ML) method with the parameters “–alrt 1000 -B 1000” and the analysis results were visualized using ITOL software (v6) (Letunic and Bork, 2019).

## Results

### Assembly and annotation of the mitochondrial genome

**Figure 1:**
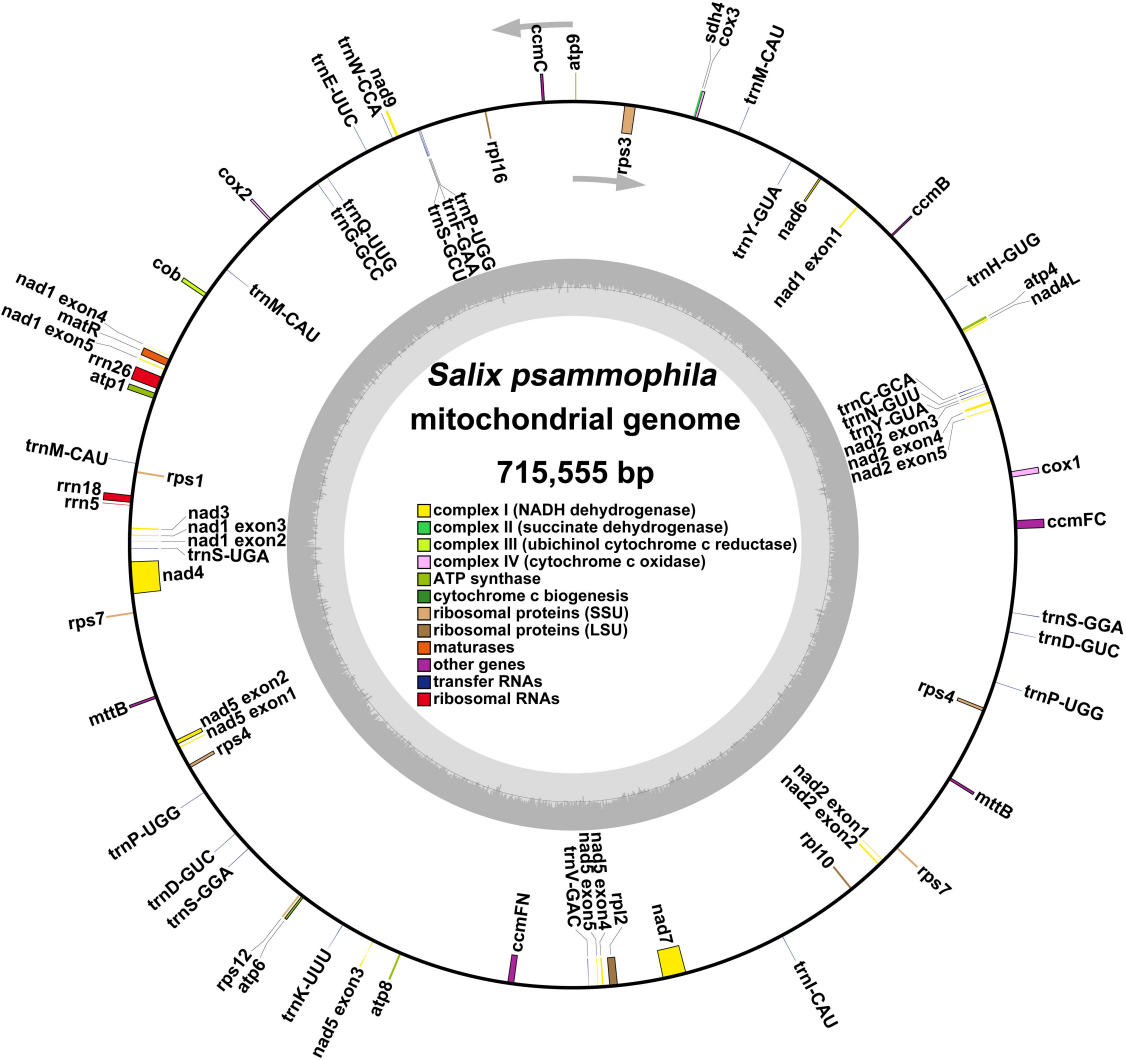
Genome circular map representing the mitochondrial genome of *S. psammophila.* The colored squares inside and outside the circular map represent various mitochondrial genes. Genes belonging to the same function are represented by the same color.

A total of 265,714 reads were utilized in assembling the *S. psammophila* mitochondrial genome, with the longest read length reaching 48,394 bp, an average read length of 18,699 bp, an N50 read length of 18,657 bp, and total read bases amounting to 4,968,733,600 bp. After exclusion of duplicated regions, one molecular circular was obtained with a total length of 715,555 bp and 44.89% GC content (**Figure 1**). We annotated the mitochondrial genome of the *S. psammophila* and the annotation results are shown in **Table 1**. A total of 33 unique PCGs were annotated, including 24 unique core genes and 9 variable genes, 18 tRNA genes (5 of which were multicopy genes), and 3 rRNA genes. The core genes include five ATP synthase genes (*atp1*, *atp4*, *atp6*, *atp8*, and *atp9*), nine NADH dehydrogenase genes (*nad1*, *nad2*, *nad3*, *nad4*, *nad4L*, *nad5*, *nad6*, *nad7*, and *nad9*), four cytochrome C biogenesis genes (*ccmB*, *ccmC*, *ccmFC*, and *ccmFN*), three cytochrome C oxidase genes (*cox1*, *cox2*, and *cox3*), one protein transport subunit gene (*mttB*), one maturases gene (*matR*), and one cytochrome b gene (*cob*). Variable genes include three RP large subunit genes (*rpl2*, *rpl10*, and *rpl16*), five RP small subunit genes (*rps1*, *rps3*, *rps4*, *rps7*, and *rps12*), and one succinate dehydrogenase (*sdh4*).

**Table 1:** Gene composition in the mitogenome of *Salix psammophila*.

Group of genes	Name of genes
ATP synthase	*atp 1*, *atp 4*, *atp 6*, *atp 8*, *atp 9*
NADH dehydrogenase	*nad 1*, *nad 2*, *nad 3*, *nad 4*, *nad 4L*, *nad 5*, *nad 6*, *nad 7*, *nad 9*
Cytochrome b	*cob*
Cytochrome c biogenesis	*ccmB*, *ccmC*, *ccmFC*, *ccmFN*
Cytochrome c oxidase	*cox 1*, *cox 2*, *cox 3*
Maturases	*matR*
Protein transport subunit	*mttB* (×2)
Ribosomal protein large subunit	*rpl2*, *rpl10*, *rpl16*
Ribosomal protein small subunit	*rps 1*, *rps 3*, *rps 4* (×2), *rps 7* (×2), *rps 12*
Succinate dehydrogenase	*sdh 4*
Ribosome RNA	*rrn 5*, *rrn 18*, *rrn 26*
Transfer RNA	*trnC-GCA*, *trnD-GUC* (×2), *trnE-UUC*, *trnF-GAA*, *trnG-GCC*, *trnH-GUG*, *trnI-CAU*, *trnK-UUU*, *trnM-CAU* (×3), *trnN-GUU*, *trnP-UGG* (×3), *trnQ-UUG*, *trnS-GCU*, *trnS-GGA* (×2), *trnS-UGA*, *trnV-GAC*, *trnW-CCA*, *trnY-GUA* (×2)

### The structure of the mitochondrial genome

**Figure 2:**
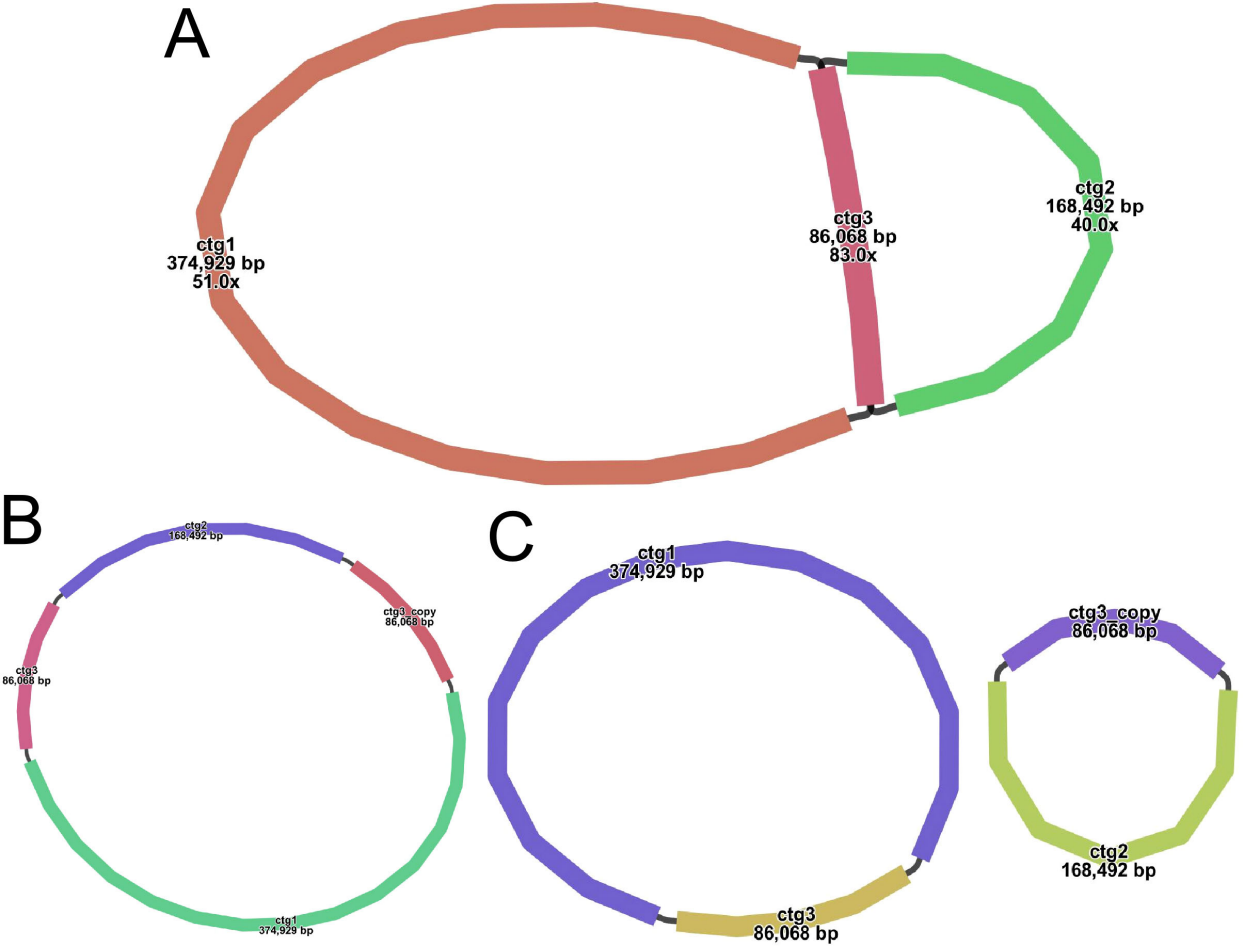
Various graphical mitochondrial genome structure maps of *S. psammophila*. **(A)** Sketch map of the mitochondrial genome assembly (node ID marked in the image). **(B)** The main configuration of the mitochondrial genome. **(C)** The minor configuration of the mitochondrial genome.

A sketch of the assembled *S. psammophila* mitochondrial genome was visualized using Bandage software (v0.8.1), and the results are shown in **Figure 2A**. The sketch contains three nodes (see **Supplementary Table S2A** for details). Each node represents a sequence (contig) obtained by assembly, and two nodes are connected by a black line, which represents the overlapping region between the two sequences. Together, all sequences form a complex multibranched closed genome structure that represents the complete mitochondrial genome sequence of *S. psammophila*. We use long reads to address several key nodes where branching exists, derive the relevant sequences that are at branching nodes, and map them to long reads. Two sequences connected by a black line appear on the same long reads and are connected at the beginning and end, which means that the long reads support the interconnection of these two sequences. A number of different connections exist on different branch nodes, but we prioritize connections that are supported by more long reads when assembling. The resolution of branching nodes due to repeated sequences (*ctg3*) based on long reads yields a circular contig sequence (**Figure 2B**), the resolution of which is summarized in **Supplementary Table S2B**. In addition, *ctg3* can be formed into a circular structure with *ctg1* and *ctg2*, respectively, to form a potential secondary conformation of the two circular (**Figure 2C**).

### Codon preference of the mitochondrial genome

Codon preference analysis was performed on 33 unique PCGs of *S. psammophila* mitochondria, and the codon usage of individual amino acids is shown in **Supplementary Table S3**. All amino acids except Met (AUG) and Trp (UGG) consist of multiple codons. Codons with relative synonymous codon use (RSCU) greater than 1 are considered to be used preferentially by amino acids. As shown in **Supplementary Figure S1**, codon usage preferences are also prevalent in mitochondrial PCGs, except for the start codons AUG and Trp (UGG), which have an RSCU value of 1. For example, the termination codon has a high codon usage preference for UAA with an RSCU value of 1.69, which is the highest among mitochondrial PCGs. This is followed by alanine (Ala), which has a high codon usage preference for GCU with an RSCU value of 1.64.

### Sequence repeat analysis

**Figure 3:**
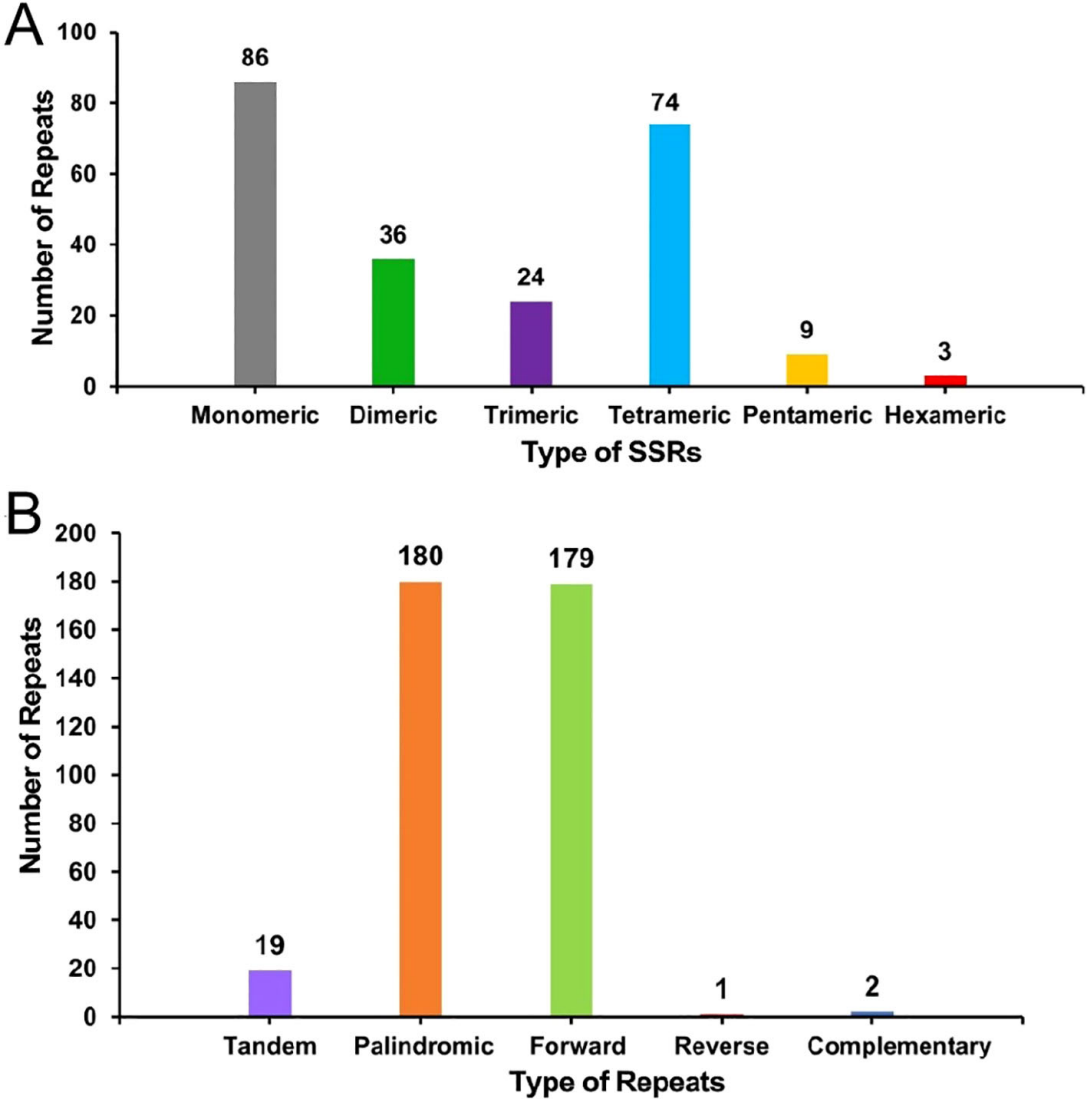
The histogram of the repeat sequence analysis. **(A)** Horizontal coordinates represent the type of SSRs, and vertical coordinates represent the number of repetitive fragments. The gray legend represents monomeric SSRs, the green legend represents dimeric SSRs, the purple legend represents trimeric SSRs, the blue legend represents tetrameric SSRs, the yellow legend represents hexameric SSRs, and the red legend represents hexameric SSRs. **(B)** The horizontal coordinates represent the type of repetitive sequence, and the vertical coordinates represent the number of repetitive fragments. The purple legend represents tandem repeats, the orange legend represents palindromic repeats, the green legend indicates forward repeats, the red legend represents reverse repeats, and the blue legend represents complementary repeats.

Repeat sequence analysis showed that a total of 232 SSRs were identified in the mitochondrial genome of the *S. psammophila*, with 86, 36, 24, 74, 9, and 3 monomers, dimers, trimers, tetramers, pentamers, and hexamers, respectively (**Figure 3A**). Among them, monomeric and dimeric SSRs accounted for 52.59% of the total SSRs. Thymine (T) monomeric repeat sequences accounted for 55.81% (48) of the 86 monomeric SSRs. Repeat sequences are categorized into tandem repeat sequences and dispersed repeat sequences. Tandem repeat sequences, also known as satellite DNA, are core repetitive units of approximately 7 to 200 bases that are repeated many times in tandem and are widely found in prokaryotic and eukaryotic genomes. There were 19 tandem repeats in the mitochondrial genes with greater than 85% match and lengths ranging from 12 to 28 bp, and 362 pairs of dispersed repeats with lengths greater than or equal to 30 bp. The dispersed repeats contained 180 pairs of palindromic repeats, 179 pairs of forward repeats and 2 pairs of complementary repeats (**Figure 3B**). Of these, the longest palindromic repeat is 260 bp and the longest forward repeat is 86,068 bp.

### Sequence transfer analysis

**Figure 4:**
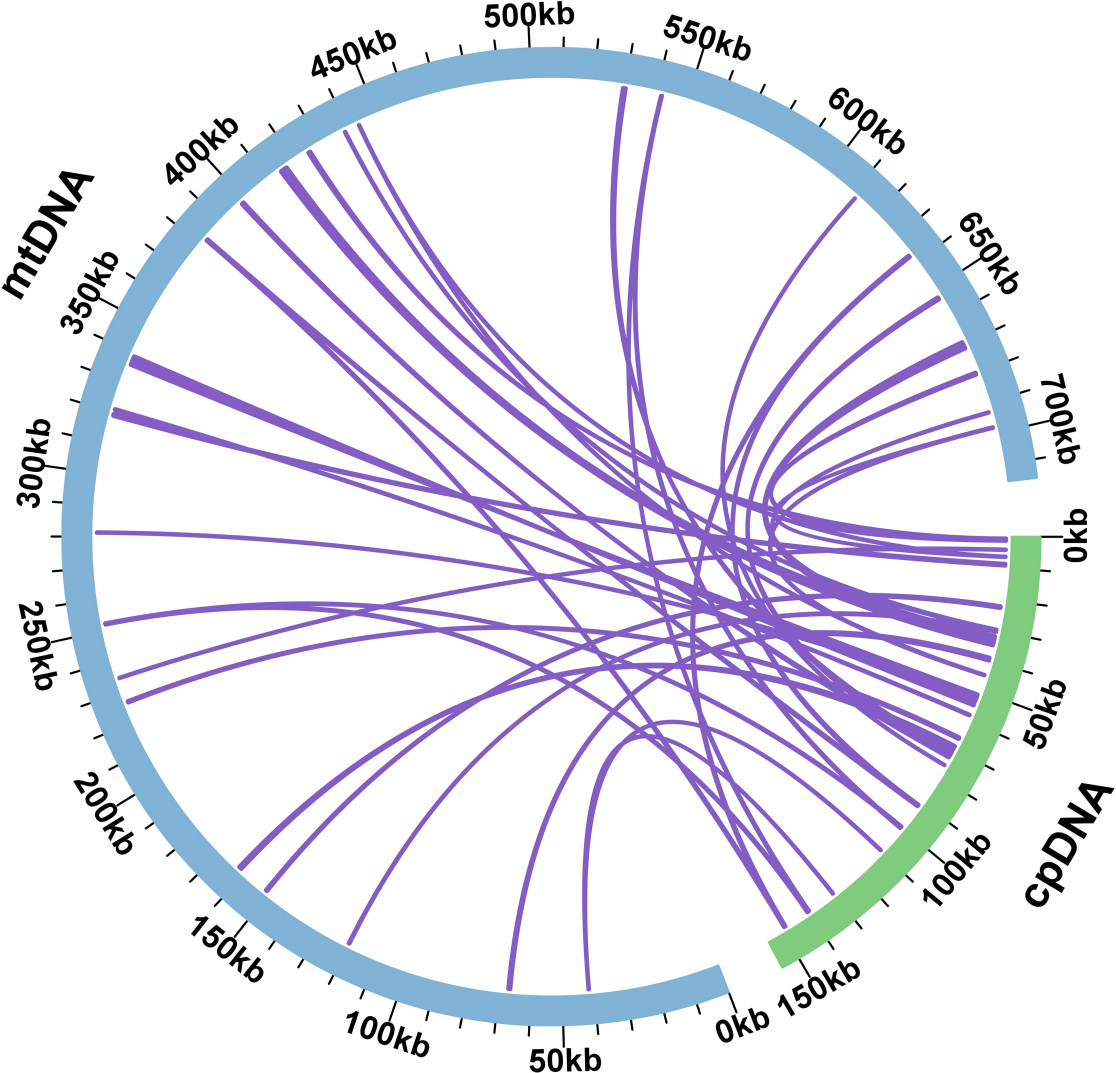
Homological sequences between mitochondrial and chloroplast genomes. The blue circular segment in the figure represents the mitochondrial genome, the green circular segment represents the chloroplast genome, and the purple line between the circular segments corresponds to a homologous fragment of the genome fragments.

During mitochondrial evolution, some fragments migrate from chloroplasts to mitochondrial genes, and the length and sequence similarity of the migrated fragments change from species to species. Based on the sequence similarity, a total of 38 fragments from the chloroplast genome were found in the mitochondrial genome of *S. psammophila*, with a total length of 16,536 bp, which accounted for 2.31% of the total length of the mitochondrial genome (**Figure 4**). Among these homologous fragments, MTPT 16 was the longest, with a length of 2,579 bp, and MTPT 8 and MTPT 9 were the shortest, both at 33 bp. Annotating these homologous sequences, we identified eight complete genes, namely, one PCG (*atpE*) and seven tRNA genes (trn*D-GUC*, *trnM-CAU*, *trnN-GUU*, *trnP-UGG*, *trnS-GGA*, *trnV-UAC*, and *trnW-CCA*).

### Prediction of RNA editing sites

**Figure 5:**
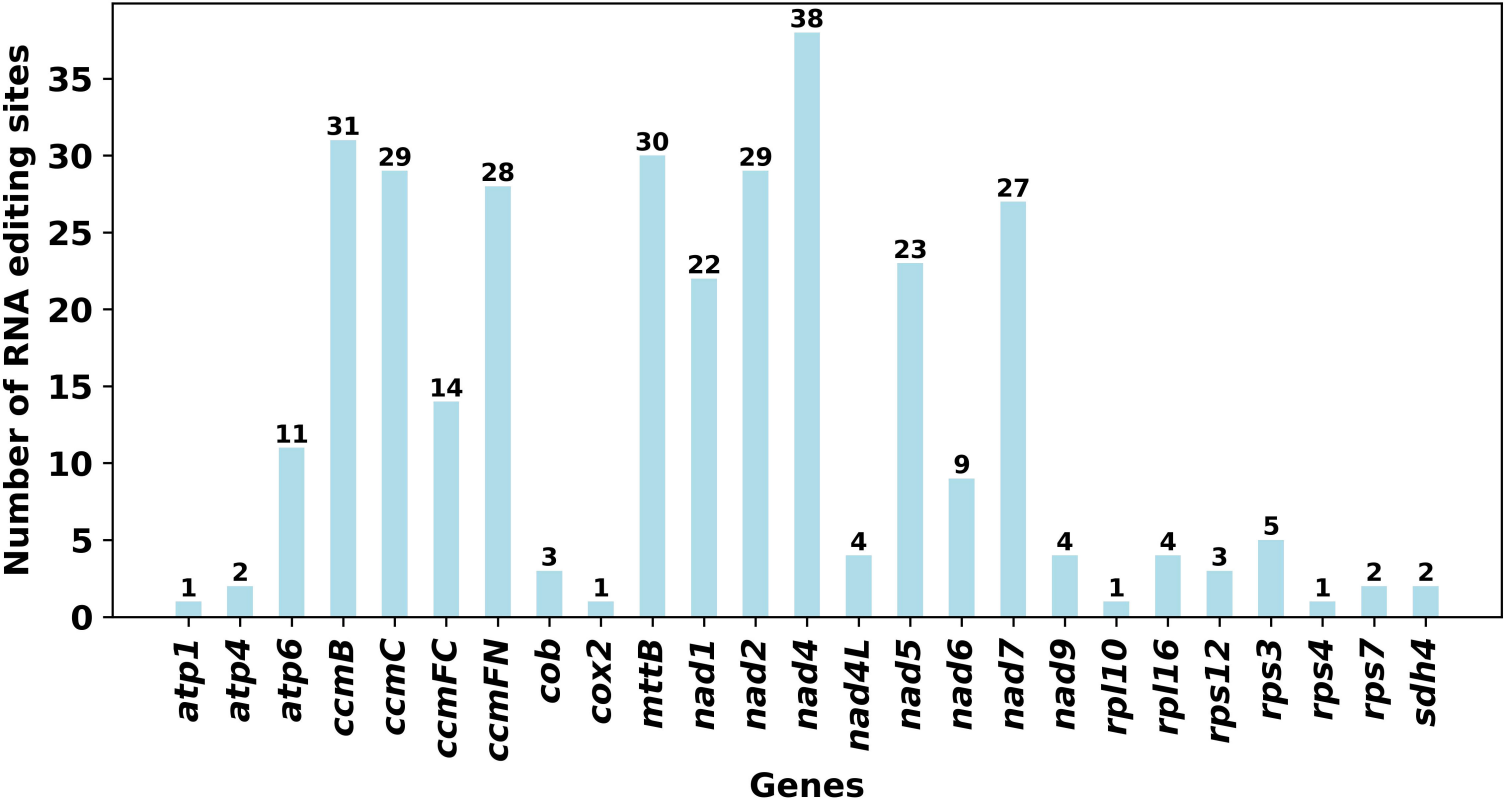
Number of RNA editing sites in all the PCGs.

RNA editing events were predicted for 33 unique PCGs of the mitochondrial genome by setting the following criterion: cutoff value = 0.9. A total of 324 potential RNA editing sites were identified, all of which were C-to-U editing and basically appeared in the second or third position, with a few occurring in the third position (**Supplementary Table S8**). As shown in **Figure 5**, among these mitochondrial genes, the most edited gene was the *nad4* gene, with 38 RNA editing sites identified. The least edited genes were *atp1*, *cox2*, *rpl10*, and *rps4*, with 1 RNA editing site identified.

### Phylogenetic analysis

**Figure 6:**
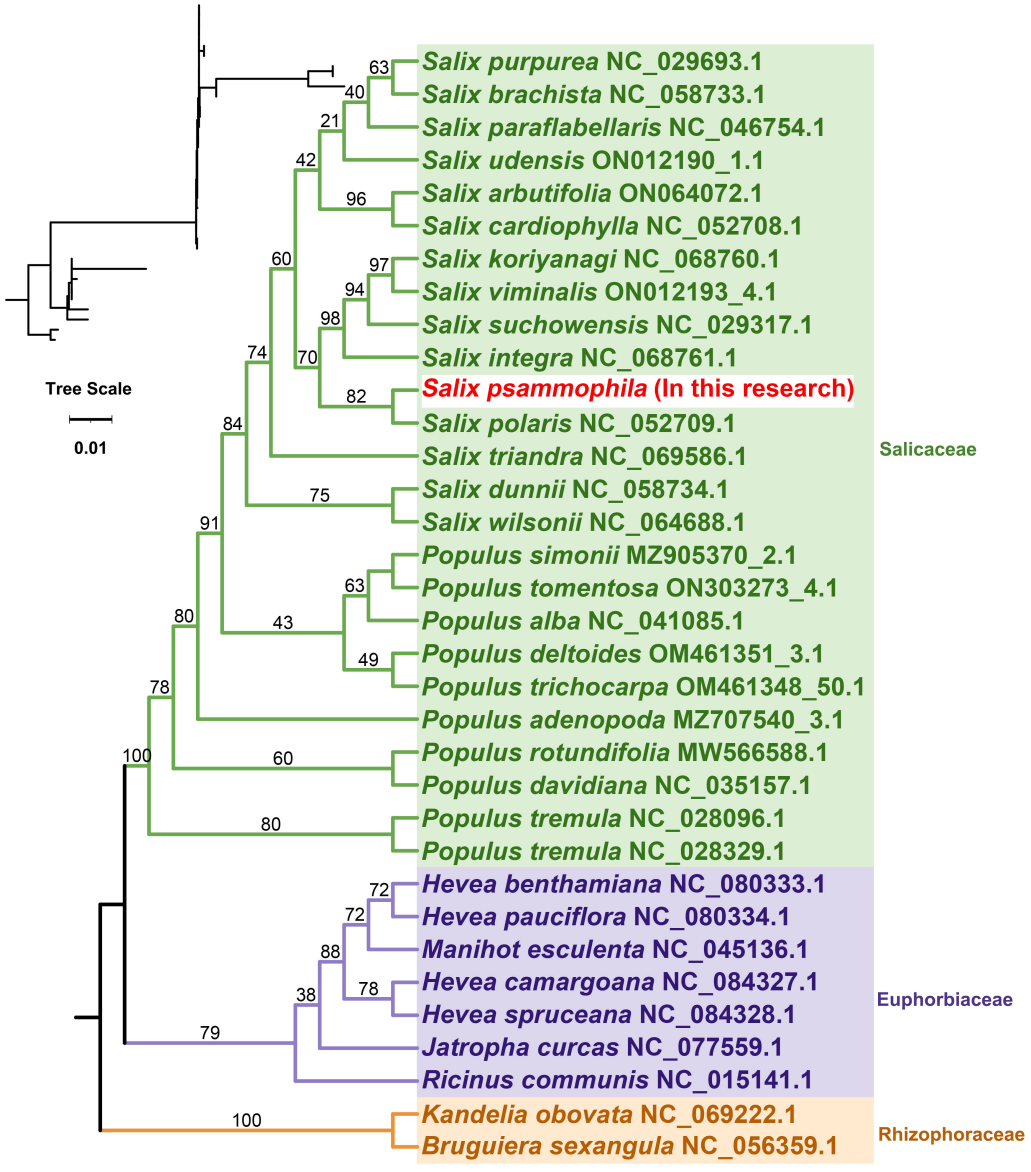
Phylogenetic relationships of *S. psammophila*.

To further explore the evolutionary relationships of the mitochondria of *S. psammophila*, phylogenetic trees were constructed for 34 species of plants from three families, namely, Salicaceae, Euphorbiaceae, and Rhizophoraceae, based on the DNA sequences of 24 conserved mitochondrial PCGs, in which *Bruguiera sexangula* and *Kandelia obovata* of the Rhizophoraceae family were set up as the outgroups (**Figure 6**). The PCGs shared by these species are *atp1*, *atp4*, *atp6*, *atp8*, *atp9*, *ccmB*, *ccmC*, *ccmFC*, *ccmFN*, *cob*, *cox1*, *cox2*, *cox3*, *matR*, *mttB*, *nad1*, *nad2*, *nad3*, *nad4*, *nad4L*, *nad5*, *nad6*, *nad7*, and *nad9*. The topology of the mitochondrial DNA phylogeny coincides with the latest classification of the Angiosperm Phylogeny Group (APG). *S. psammophila* belongs to the family Salicaceae in the order Salicales and is most closely related to *S. polaris.*

### Synteny analysis

**Figure 7:**
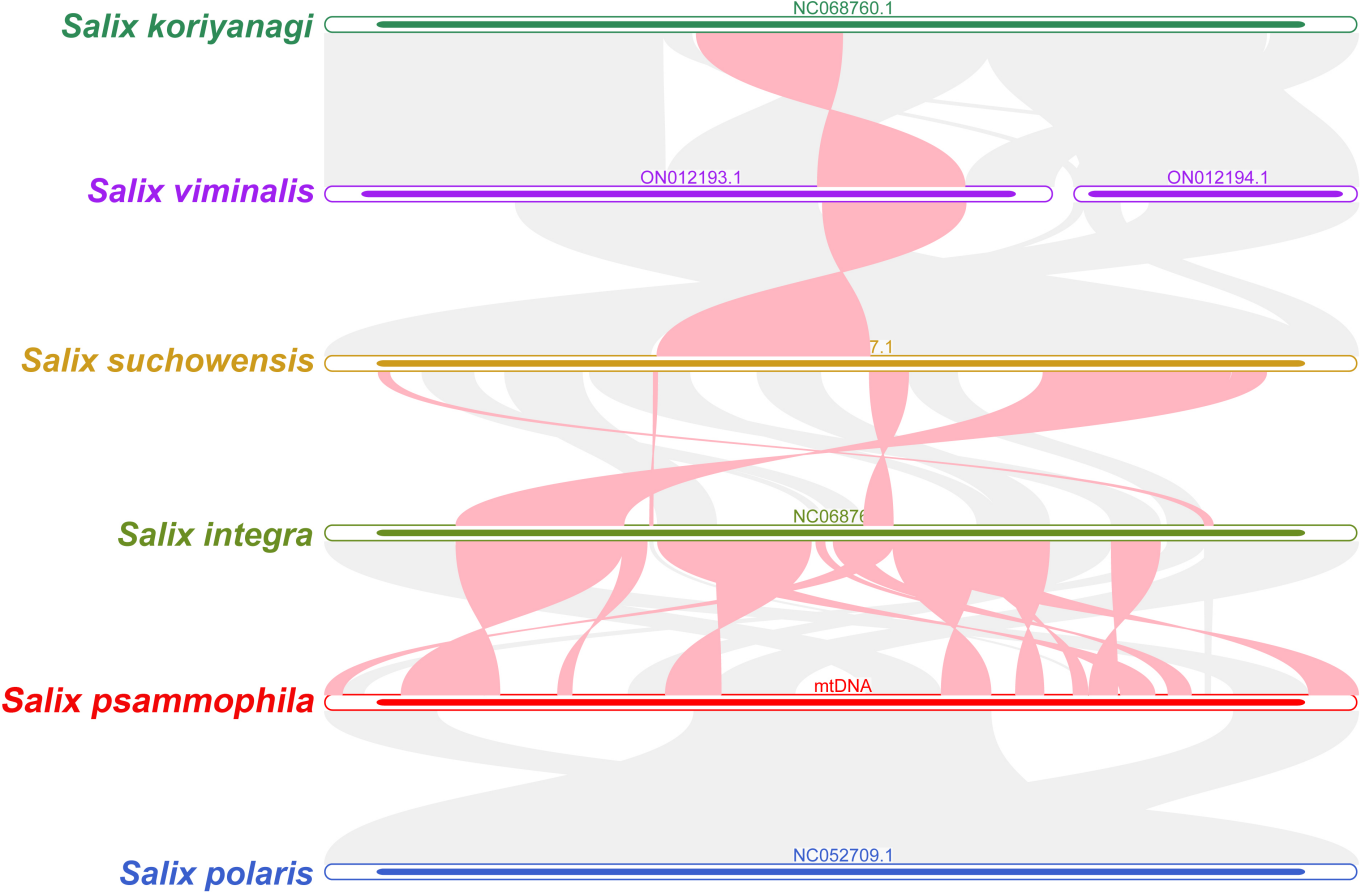
Synteny analysis of six different species in the genus Salix. The red curved regions indicate regions where inversion occurs, and the gray regions indicate regions of good homology.

*S. koriyanagi*, *S. viminalis*, *S. suchowensis*, *S. integra*, *S. psammophila*, and *S. polaris* were selected for the synteny analysis. As shown in **Figure 7**, there are a large number of homologous collinear blocks between the mitochondrial genomes of *S. psammophila* and the other five willow species. In addition, there are some gap regions where sequences are species-specific and have no homology with the rest of the species. The results showed that the mitochondrial genome of *S. psammophila* underwent genomic rearrangement with *S. integra* but not with *S. polaris*.

## Discussion

### Complexity and variability mitochondrial genome size and structure in plants

The plant mitochondrial genome is an evolutionary dynamic entity with great plant diversity in terms of size, structure, gene content, etc (Petersen et al., 2020). *S. psammophila* mitochondrial genome is a circular structure with a total length of 715,555 bp and a GC content of 44.89%. The mitochondrial genome structure and GC content were similar to those of the known *P. deltoides*, *P. simonii*, *S. wilsonii*, and *S. suchowensis*, all of which were cyclic in structure, and the GC content was approximately 44.80%. However, there are also some differences in the genome structure and size of the Salicaceae plants. The mitochondrial genomes of both *P. deltoides* and *P. simonii* are three circulars, the *P. tremula*, *P. tremula × P. alba*, *P. alba*, and *P. davidiana* are the typical single circular sequences, as are the *S. suchowensis, S. wilsonii*, and *S. purpurea* (Kersten et al., 2016; Choi et al., 2017; Ye et al., 2017; Brenner et al., 2019; Chen et al., 2020; Bi et al., 2022; Han et al., 2022; Qu et al., 2022). In the genus *Populus*, the mitochondrial genome size is very conserved, and poplars such as *P. simonii* (781,478 bp), *P. deltoides* (802,637 bp), *P. tremula* (783,442 bp), and *P. alba* (838,420 bp) are essentially similar in mitochondrial genome size (Kersten et al., 2016; Brenner et al., 2019; Bi et al., 2022; Qu et al., 2022). Numerous studies have shown that a large number of repetitive units, the incorporation of foreign sequences, and the acquisition or loss of large genetic fragments of their own are the main causes of large differences in plant mitochondrial genomes (Wu et al., 2022). In the mitochondrial genome of the genus *Populus*, there are no repetitive sequences >350 bp, which may be the reason for the small differences in size and structure of the genus *Populus* mitochondrial genome (Bi et al., 2022). In contrast, the mitochondrial genome size of the genus Salix is highly variable, ranging from 599 kb (*S. purpurea*) to 735 kb (*S. cardiophylla*) (Wei et al., 2016; Chen et al., 2020). The size of plant mitochondrial genomes varies dramatically between families and even between different species of the same genus. For example, the mitochondrial genome size difference between woody cotton (*Gossypium arboreum*) and upland cotton (*Gossypium hirsutum*) is 65.6 kb (Ala et al., 2023). Furthermore, there was no positive correlation between genome size and gene number; *P. deltoides* mitochondrial genomes had the largest size but not the largest number of genes, with the largest number of genes being found in *S. brachista*, and the difference in the number of genes between their mitochondrial genomes was small (**Supplementary Table S11**). The mitochondrial genome of *Welwitschia mirabilis* is more than twice the size of *Cycas taitungensis*, but had fewer genes than *Cycas taitungensis* (Guo et al., 2016), Meanwhile, the sizes of the *S. psammophila* and *S. wilsonii* mitochondrial genomes are similar, and there are significant differences in the types and copy numbers of tRNA, suggesting that different species within the genus *Salix* possess their unique biological functions. Previous studies have demonstrated that the phylogeny of the mitogenome in *Populus* can effectively distinguish between different groups of poplar trees (Qu et al., 2022). Similarly, species within the genus *Salix* can also be categorized using phylogenetic trees. For instance, *S. wilsonii* and *S. dunnii* are classified under Sect. Wilsonianae, while *S. brachista* and *S. paraflabellaris* are grouped under Sect. Lindleyanae.

### Repeat sequences in the mitochondrial genome of Salicaceae plants

A large number of repetitive sequences are distributed in the plant mitochondrial genome, and their size and arrangement are closely related to the differences and rearrangements among the plant mitochondrial genomes, which play an important role in the evolution of plant mitochondria (Tanaka et al., 2014; Wynn and Christensen, 2019; Yang et al., 2021). Repetitive sequences in plant mitochondrial genomes can be categorized into two groups, dispersed repeats and tandem repeats (Wu et al., 2019). Simple sequence repeat (SSR) is a highly variable tandem repeat sequence of 1–6 bp, commonly used as a molecular marker for species identification and genetic diversity studies (Ma et al., 2017; Li and Ye, 2020). A total of 232 SSRs were detected in the *S. psammophila* mitochondrial genome, and the analysis revealed that 95% of the mononucleotide SSRs were A/T, which was similar to the mitochondrial genomes of other genus Salix, with the highest proportion of A/T repeats among the mononucleotide repeats (Ye et al., 2017; Han et al., 2022). In addition to tandem repeat sequences, plant mitochondrial genomes are characterized by the presence of a large number of dispersed repeat sequences, including palindromic repeat sequences, forward repeat sequences, reverse repeat sequences, and complementary repeat sequences. There are 362 dispersed repeat sequences in the *S. psammophila* mitochondrial genome, with palindromic and forward repeats being the most abundant. As shown in **Supplementary Table S6**, a total of 236 (65.19%) dispersed repeats range from 30 to 39 bp, 108 (29.83%) repeats are between 40 and 99 bp in length, 4.7% of the repeats are within the range of 100 to 300 bp in length, and one of the repeats is 86,068 bp, which is a forward repeat. Larger repetitive sequences are critical in genomic structural changes, and paired forward and reverse repeat sequences (>1 kb) can produce subgenomic or isomeric conformations (Bi et al., 2020). In addition to *S. psammophila*, *S. cardiophylla*, *S. paraflabellaris*, and *S. suchowensis* mitochondrial genomes also contain repetitive sequences greater than 1 kb in length, and their impact on the structure and size expansion of mitochondrial genomes is of interest (Chang et al., 2013; Dong et al., 2018; Chen et al., 2020).

### The *rps14* function during the evolution of the genome of Salicaceae plants

The PCG analysis based on the mitochondrial genome of *S. psammophila* and *S. suchowensis*, *S. wilsonii*, *P. simonii*, and *P. deltoides* revealed that the mitochondrial genome of Salicaceae plants, like other plants, is relatively conserved. It has been shown that the number of PCGs gradually decreases during the evolution of species, and that the genes lost in most cases are RP genes and succinate dehydrogenase genes (Adams and Palmer, 2003). Through comparative analysis of the mitochondrial genomes of several Salicaceae plants, it was found that the *rps14* gene was lost in *S. psammophila*, *S. suchowensis*, *S. wilsonii*, and *P. simonii* (Ye et al., 2017; Bi et al., 2022; Han et al., 2022; Qu et al., 2022). RPs are proteins in ribosomes that play a crucial role in ribosome biogenesis, protein synthesis, cell growth, development, and apoptosis (Ramakrishnan and White, 1998; Naora and Naora, 1999; Maguire and Zimmermann, 2001; Wang et al., 2013b). *S. psammophila* and *P. simonii* play important roles in resisting wind damage and fixing sand dunes and have excellent characteristics such as drought resistance, tolerance to barrenness, and strong adaptability. They are the main tree species in the Northwest and North China regions (Jia et al., 2019; Wu et al., 2020; Yang et al., 2022). *S. suchowensis* is mainly distributed in central and eastern China, and has the function of windbreak and sand fixation (Jia et al., 2020; Wang et al., 2024). The original ecological area of *P. deltoides* is from the southeastern United States to southern Canada and plays an important role in riparian ecosystems (Fahrenkrog et al., 2017). Based on their different geographical distribution and physiological characteristics, we speculate that the loss of *rps14* in the evolution of Salicaceae plants may lead to their sensitivity to adversities such as drought (**Supplementary Table S12**). RPs are important for the translation of diverse proteins and are involved in overall cellular adaptation under stress conditions (Vemanna et al., 2019). It has been shown that 46 RPs are downregulated for expression under drought conditions (Narsai et al., 2013). Bi et al. found that *rps14* and the *ccmB*, *ccmFC*, *rps1*, and *rps10* genes may play an important role in the evolutionary process by analyzing selection pressures on PCGs of the *Phaseolus vulgaris* mitochondrial genome (Bi et al., 2020).

### Conservation of RNA editing events in plant mitochondrial genomes

The oxidative phosphorylation system in mitochondria consists of five complexes (I–V), the normal assembly of which requires standard processing of pre-mRNAs, involving RNA editing and intron shearing, and is important for the maintenance of mitochondrial functions (Dudkina et al., 2006; Li et al., 2014; Sun et al., 2018). RNA editing is a phenomenon in which genetic information is altered through the insertion of nucleotides, or nucleotide deletions and transitions, into the messenger RNA of a functional gene (Keller et al., 1999). RNA editing can occur in a variety of forms, including C-to-U, U-to-C, and A-to-I conversions; U insertions and deletions; and G insertions (Small et al., 2020). In plants, RNA editing events occur mainly in plant mitochondria and chloroplasts, and take place mainly in the form of a C-to-U transition (Hao et al., 2021). We analyzed the 33 PCGs within the mitochondrial genome of *S. psammophila* and identified a total of 324 RNA editing sites. All of these sites exhibited C to U transitions, which is consistent with findings in *S. suchowensis* and *P. deltoides* (Ye et al., 2017; Qu et al., 2022). In addition, we found the highest number of edits to *nad4* in *S. psammophila* and *S. suchowensis* mitochondria. It has also been found in plants such as *Corydalis saxicola*, with the most editing of the *nad4* in the mitochondrial genome (Maldonado et al., 2022; Li et al., 2024). The *nad4* is one of the components of complex I, which is the largest complex related to respiration in the mitochondria of land plants and is important for maintaining plant growth and development (Møller et al., 2021). Thus, extensive editing of the *nad4* may be important for the functioning of complex I in plant mitochondria.

## Conclusions

In this study, we successfully assembled and annotated the mitochondrial genome of *S. psammophila* for the first time, identifying it as a single circular structure with a total length of 715,555 bp and a GC content of 44.89%. Comparative analysis with mitochondrial genomes of *S. wilsonii*, *S. suchowensis*, *P. simonii*, and *P. deltoides* revealed that PCGs within the Salicaceae family are highly conserved, with significant variations primarily observed in RPs. Notably, *rps14* is absent in *S. psammophila*, a species well-adapted to arid environments. This absence might indicate a role in enhancing tolerance to environmental stresses such as drought. These findings contribute valuable insights into the phylogenetic and genetic research of Salicaceae plants.

## Data Availability

The datasets presented in this study can be found in online repositories. The names of the repository/repositories and accession number(s) can be found in the article/**Supplementary Material**.

## Supplementary material

Supplementary Figure 1 The codon preference analysis of the mitochondrial genome. Different colors of the same amino acid represent the RSCU values of different codons editing this amino acid.
