# Supplementary figures and images for: Assembly and comparative analysis of the first complete mitochondrial genome of Salix psammophila, a good windbreak and sand fixation shrub
Source: Front Plant Sci. 2024 Oct 2;15:1411289. doi: 10.3389/fpls.2024.1411289 (PMC11479937; doi:10.3389/fpls.2024.1411289)

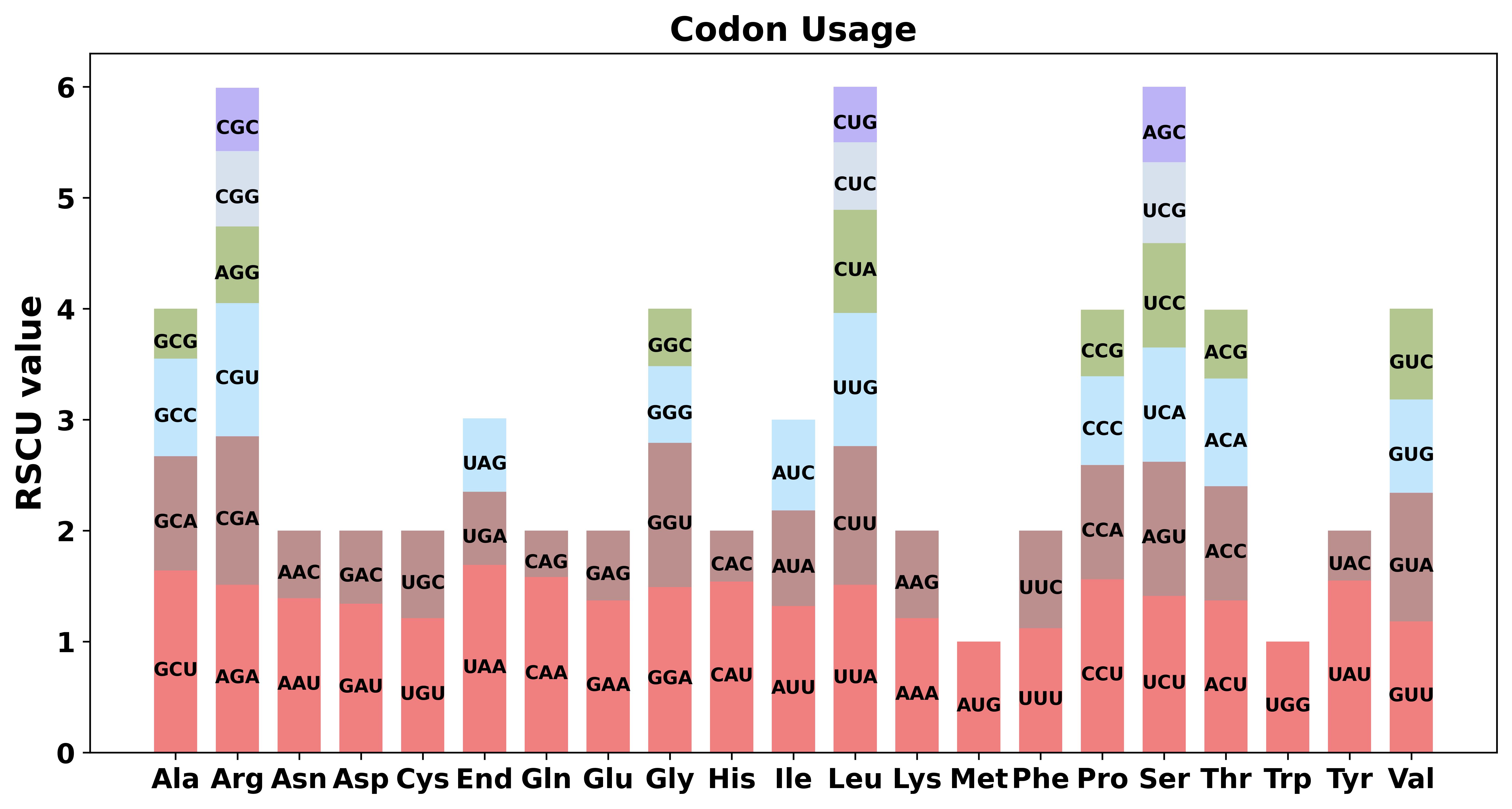

Supplement: Supplementary Figure 1 — The codon preference analysis of the mitochondrial genome. Different colors of the same amino acid represent the RSCU values of different codons editing this amino acid. [file DataSheet1.zip › Figure S1.JPEG]
